# Management of Chronic Uncontrolled Diabetes With Ketoacidosis and Hyperosmolar Hyperglycemic State

**DOI:** 10.7759/cureus.92913

**Published:** 2025-09-22

**Authors:** Sunil Dwivedi, Anupam K Sriwastava, Indu Saxena, Manoj Kumar

**Affiliations:** 1 Department of Biochemistry, NKP Salve Institute of Medical Sciences and Research Centre and Lata Mangeshkar Hospital, Nagpur, IND; 2 Department of Biochemistry, All India Institute of Medical Sciences, Gorakhpur, IND; 3 Department of Physiology, Maharshi Vashishtha Autonomous State Medical College (MVASMC), Rampur, IND

**Keywords:** diabetes mellitus, diabetic ketoacidosis, glycemic control, hyperglycemia, hyperosmolar hyperglycemic state

## Abstract

Diabetes mellitus (DM) continues to pose a significant global health burden. Acute complications of diabetes are life-threatening conditions: diabetic ketoacidosis (DKA) and hyperosmolar hyperglycemic state (HHS). DKA predominantly affects young and type 1 diabetic patients. It is characterized by metabolic acidosis and ketonemia. Older patients with type 2 diabetes are at a greater risk of developing HHS, which is marked by profound hyperglycaemia and hyperosmolarity without significant ketoacidosis.

This review highlights the integrative approach needed for effective management. Since both conditions share certain common features, it is important to distinguish between them and initiate appropriate treatment, including prompt fluid resuscitation, insulin therapy, and electrolyte correction. Identification and treatment of precipitating factors is vital. Post-acute care strategies include medical stabilization as well as long-term behavioral modifications, like structured diabetes education, psychosocial support, and implementation of technology-driven interventions (continuous glucose monitoring and telemedicine) to reduce recurrence. HHS has up to 20% higher mortality due to delayed diagnosis and comorbidities, while DKA’s acute fatality remains comparatively lower at 1-5%. Therefore, early identification, aggressive management, and coordinated long-term follow-up are essential. A shift from episodic care to proactive, patient-centered disease management may significantly improve outcomes and quality of life for individuals with chronic diabetes.

## Introduction and background

Diabetes mellitus (DM) is a chronic metabolic disorder characterized by hyperglycemia due to impaired insulin secretion, action, or both [1]. Both diabetic ketoacidosis (DKA) and hyperosmolar hyperglycemic state (HHS) may occur in patients with either type 1 or type 2 diabetes. DKA is characterized by the presence of hyperglycemia or a prior history of diabetes, ketonemia, and metabolic acidosis with increased anion gap. The diagnosis of HHS is based on the following criteria: hyperglycemia (plasma glucose > 600 mg/dL), hyperosmolality, absence of significant ketonemia, and absence of acidosis.

In the past 10 years, the incidence of DKA has increased by 30%, causing more than 140,000 admissions per year in the U.S. alone [2]. DKA and HHS are emergencies that can lead to morbidity and mortality if not promptly recognized and treated. This review aims to synthesize recent evidence on management strategies and long-term approaches.

## Review

Methods

This narrative review focuses on two diabetic complications: DKA and HHS. It briefly describes the epidemiology, pathophysiology, risk factors, clinical presentation, diagnosis, complications, and management of these two conditions. We searched PubMed, Google Scholar, and Cochrane for studies from 2000 to 2025 using the terms DKA, HHS, and uncontrolled diabetes. Facts related to metabolism were selected from articles published earlier. No pooled statistical analyses were performed.

Epidemiology of DKA and HHS

The global rise in diabetes has been accompanied by a growing burden of uncontrolled diabetes and its acute complications. The conditions of DKA and HHS may co-exist in some cases. HHS carries a higher mortality rate (up to 20%) compared to DKA (1-5%). A German study involving 31,330 patients with type 1 diabetes reported a DKA incidence of 4.81 per 100 patient-years, with higher risk among adolescents, females, those with elevated HbA1c, and longer disease duration [3]. In the case of HHS, an inpatient mortality of 5-16% has been reported [2]. Prognosis in HHS is affected by factors such as dehydration, co-existing illnesses, and older age. Patients with recurrent HHS episodes face a significantly elevated risk of death, with hazard ratios of 2.8 (for one episode) and 4.5 (for multiple episodes) compared to those reporting no previous episodes [4]. The U.S. national data from 1990 to 2010 showed a reduction in deaths from hyperglycemic crises, dropping to 2.7 cases per 10,000 [5]. According to the Youth Diabetes Registry (YDR) in India, around 28.7% of young individuals with type 1 diabetes present with DKA at diagnosis, slightly lower than the 35.3% reported in the U.S. SEARCH Registry [6].

While DKA is more common in insulin-dependent type 1 diabetes mellitus (T1DM), it also affects adults with non-insulin-dependent type 2 diabetes mellitus (T2DM). A study in Maharashtra found that 80% of DKA cases occurred in people with type 2 diabetes [7,8]. However, pediatric centers have reported significantly lower mortality rates, such as 11.5% in a tertiary hospital in South India [9]. According to one study, the in-hospital mortality rate for DKA reached 39.6% during the COVID-19 pandemic [10]. HHS remains underreported in India but is a serious concern, particularly among elderly individuals with type 2 diabetes.

Pathophysiology

**Figure 1:**
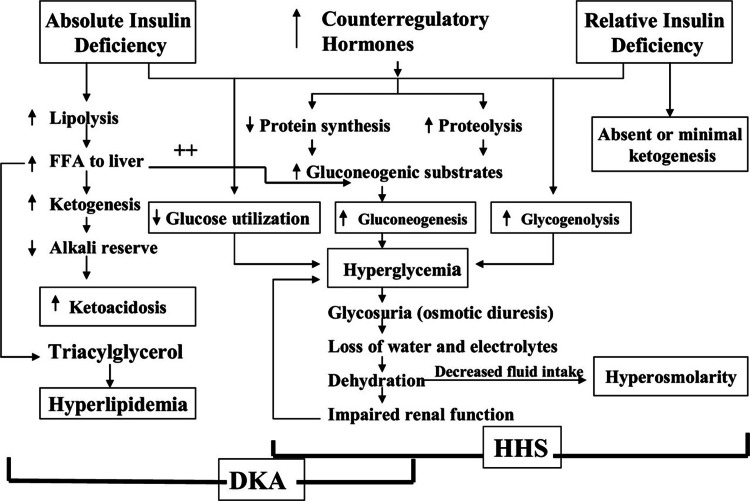
Pathophysiologic mechanisms for diabetic ketoacidosis and hyperosmolar hyperglycemic state FFA: free fatty acid; DKA: diabetic ketoacidosis; HHS: hyperosmolar hyperglycemic state Image from John Wiley and Sons and Copyright Clearance Center, with permission (license number: 6027620725584)

Pathophysiologic mechanisms for the development of DKA and HHS include a significant deficiency of insulin and increased concentration of the hyperglycemic hormones such as glucagon, catecholamines, growth hormone, and cortisol (Figure 1). The release of cytokines in both conditions produces a decrease in the response to insulin.

DKA

DKA occurs due to an absolute insulin deficiency with contributions from the counter-regulatory hormones (glucagon, cortisol, catecholamines, and growth hormone). The hormone-sensitive lipase (HSL) present in adipocytes, upon activation by increased glucagon and relative insulin deficiency, hydrolyzes stored triglycerides to fatty acids and glycerol [11]. The free fatty acids (FFAs) are taken up by the liver and muscles. Extra fatty acids are converted to ketone bodies in the hepatocytes, a process predominantly stimulated by glucagon [12,13] and increased glucagon/insulin ratio [14]. The production of excess ketone bodies uses the alkali reserve in blood, causing ketoacidosis. Enhanced gluconeogenesis and glycogenolysis and the loss of glucose uptake by adipocytes and myocytes exacerbate hyperglycemia. The entry of FFAs into the mitochondrial matrix for beta oxidation depends on carnitine acyl transferase (CAT)-1. Malonyl-CoA, the initial intermediate of fatty acid biosynthesis, is a potent inhibitor of CAT-1. When the glucagon/insulin ratio is high, the level of malonyl-CoA falls, removing the inhibition from CAT-1. FFAs are rapidly transported into the mitochondrial matrix, undergo beta oxidation to form acetyl-CoA, the substrate for ketogenesis.

HHS

Hyperglycemia with hyperosmolarity, but without significant ketoacidosis, is a characteristic of HHS. In spite of the presence of insulin in the blood, insulin resistance prevents the entry of glucose into adipocytes and myocytes due to the deficiency of insulin-dependent glucose transporter GLUT-4 on the cell membrane. Although insulin resistance is sufficient to cause hyperglycemia, enough insulin is present to lower the levels of counter-regulatory hormones in the blood and to inhibit lipolysis. Thus, unregulated ketogenesis does not occur. The residual insulin activity is a key mechanism preventing significant ketosis, unlike in DKA [15]. Hyperglycemia-induced osmotic diuresis results in dehydration. Hyperglycemia and dehydration together contribute to the increased blood osmolarity, resulting in HHS without any significant ketoacidosis. During the COVID-19 pandemic, the incidence of both DKA and HHS was reportedly increased [10].

Risk Factors

DKA is often the initial presentation of T1DM. Some patients with undiagnosed type 2 diabetes may be diagnosed with DKA or with mixed DKA/HHS. Both DKA and HHS may be precipitated by infection (e.g., of the urinary tract or lungs) and medical or surgical emergencies (myocardial infarction, stroke, pancreatitis, or trauma). Medicines like glucocorticoids (e.g., prednisone, methylprednisolone), thiazides (e.g., hydrochlorothiazide), and antipsychotics (e.g., olanzapine, clozapine), and substance abuse, including alcohol and cocaine, also increase the risk of hyperglycemia with DKA or HHS [16-20]. Euglycemic diabetic ketoacidosis (EDKA) is a rare complication of sodium-glucose cotransporter-2 inhibitors (SGLT-2i) that are used in the treatment of T2DM. EDKA may occur in both T1DM and T2DM and is characterized by euglycemia (blood glucose < 250 mg/dL) with metabolic acidosis (arterial pH < 7.3; serum bicarbonate < 18 mEq/L). The use of SGLT-2i in T1DM is recommended with extreme caution. Up to 20% of recurrent DKA episodes in young patients were reportedly associated with psychological risk factors like eating disorders and depression. Malfunction of the insulin pump is also a cause of DKA [21,22]. Inadequate insulin dose and poor adherence to treatment may also cause DKA or HHS [23,24]. Barski et al. have described the rise in the development of euglycemic-DKA in persons using SGLT-2i. Latent autoimmune diabetes of adulthood (LADA), insulin withdrawal or dose reduction, surgery, or acute medical illness, and very low-carbohydrate diets are also risk factors for the development of DKA [25]. Table 1 below compares the risk factors for DKA and HHS.

**Table 1:** Risk factors for DKA and HHS UTI: urinary infection; MI: myocardial infarction; T2DM: type 2 diabetes mellitus; T1DM: type 1 diabetes mellitus; DKA: diabetic ketoacidosis; HHS: hyperosmolar hyperglycemic state; SGLT-2i: sodium-glucose cotransporter-2 inhibitors

Risk factor	DKA	HHS	References
Type of diabetes	Common in type 1 DM, also in T2DM	Primarily in type 2 DM	Kitabchi et al., 2009 [25]
Infection (UTI, pneumonia, sepsis)	Most common precipitating factor	Most common precipitating factor	Garg et al., 2004 [20]
Noncompliance with insulin/oral meds	Very common, especially in youth	Common in the elderly or with cognitive decline	Kiran, 2022 [9]
New-onset/undiagnosed diabetes	Often seen, especially in T1DM	Seen in elderly or late-onset T2DM	Praveen et al., 2021 [5]
Poor socioeconomic status/access to care	Significant factor in developing countries	Contributes to delayed diagnosis	SP et al., 2015 [8]
Physical stress (MI, trauma, stroke)	Can trigger DKA	Strongly associated with HHS	Kitabchi et al., 2009 [25]
Alcohol or drug use (e.g., cocaine)	Recognized trigger	Rare, but possible	Barski et al., 2019 [24]
Certain medications (e.g., steroids, SGLT-2i)	SGLT-2 inhibitors linked to euglycemic DKA	Diuretics, antipsychotics may contribute	Umpierrez et al., 2024 [15], Barski et al., 2019 [24]
Age group	Mostly young adults/adolescents	Mostly elderly (>60 years)	Garg et al., 2004 [20]

Clinical presentation

DKA

Clinical presentation of DKA in undiagnosed T1DM patients is abdominal pain with nausea/vomiting, Kussmaul respiration, fruity breath odor due to acetone, and dehydration. The onset of symptoms is rapid. Severity of symptoms may vary, with some patients reporting only fatigue, headache, and poor appetite, while others may also show signs of confusion. Many newly diagnosed T1DM patients report a brief period of fatigue and classic symptoms of hyperglycemia, like polyuria, polydipsia, and weight loss, preceding the DKA episode. Diffuse abdominal pain is reported in 46% of patients, and nausea and vomiting in up to two-thirds of patients. About half the patients present with lethargy and stupor, but less than 25% present with loss of consciousness [2].

HHS

The typical patient with HHS is older than 60 years of age with an infection or acute illness, who has delayed seeking medical attention. The onset of symptoms is gradual, over a period of several days/weeks. Characteristic signs like neurological deficits (e.g., confusion, drowsiness, loss of vision, hallucinations, hemiparesis, seizures) are common due to hyperosmolarity. The majority of patients with HHS present with a history of malaise, weakness, blurred vision, dehydration, and progressive decline in mental status. Dehydration is apparent on physical examination, with dry mucous membranes, poor skin turgor, and/or hypotension [24]. Osmotic symptoms (dehydration, tachycardia, and hypotension) often precede an acute episode. HHS has a high risk of thromboembolic events due to increased blood viscosity.

Diagnosis

The diagnosis of DKA is based on the triad of hyperglycemia (blood glucose > 200 mg/dL) or a prior history of diabetes, irrespective of the current reading, ketonemia (venous or capillary β-hydroxybutyrate ≥3.0 mmol/L or urinary ketone 2+ or higher), and metabolic acidosis (pH < 7.3 or bicarbonate concentration < 18 mmol/L) [26]. Anion gap is usually > 12; however, nausea, vomiting, and renal losses can increase this through loss of bicarbonate. Kussmaul breathing produces respiratory acidosis, often resulting in a mixed metabolic and respiratory acid-base disturbance. In such a case, blood pH may not be as low as 7.3. Measurement of serum β-hydroxybutyrate is preferred instead of the nitroprusside test for acetone [27].

HHS diagnosis is based on the following criteria: hyperglycemia (plasma glucose > 600 mg/dL), hyperosmolality (total serum osmolality > 320 mOsm/kg), absence of significant ketonemia (β-hydroxybutyrate < 3.0 mmol/L or urine ketone < 2+), and absence of acidosis (blood pH ≥ 7.3 and bicarbonate concentration ≥ 15 mmol/L). The diagnostic differences are summarized in Table 2.

**Table 2:** Diagnostic differences between DKA and HHS DKA: diabetic ketoacidosis; HHS: hyperosmolar hyperglycemic state

Parameter	DKA	HHS
Blood glucose (mg/dL)	>250	>600
Arterial pH	<7.3	>7.3
Serum bicarbonate (mEq/L)	<18	>18
Serum ketones	Present	Minimal or absent
Effective serum osmolality	Variable	>320 mOsm/kg
Anion gap	Increased	Normal or slightly elevated
Mental status	Alert to stupor/coma (depending on severity)	More commonly stupor/coma due to hyperosmolality

Other laboratory tests

In both cases, i.e., DKA and HHS, serum urea and creatinine levels should immediately be obtained to assess renal function. Monitoring of serum potassium levels is crucial at the time of diagnosis as well as during treatment. Electrolyte abnormalities (notably hypokalemia), cerebral edema, and cardiac arrhythmias are particularly dangerous in pediatric patients [28]. A white blood cell count of >25,000/μL may indicate an underlying infection and requires further investigation to diagnose the underlying cause. 

Appraisal of Laboratory Findings

One common pitfall is pseudohyponatremia, where hyperglycemia induces an osmotic shift of water into the extracellular space, diluting sodium levels. This can result in falsely low sodium concentrations unless corrected for hyperglycemia using established formulas. Nitroprusside-based tests underestimate total ketones in DKA, as they detect acetoacetate but not the predominant β-hydroxybutyrate. If clinical suspicion of DKA is high, a negative urine dipstick for ketones does not exclude DKA. Furthermore, in HHS, serum osmolality may be underappreciated if only calculated osmolality is considered (ignoring contributions from other osmotically active substances like ethanol or mannitol), leading to misdiagnosis. Additionally, lab measurement of potassium can be misleading, as initial hyperkalemia due to acidosis and insulin deficiency may mask significant total body potassium depletion, which only becomes evident after insulin therapy and correction of acidosis [26,29].

Differential diagnosis

Some common pathologies may mimic DKA. These include starvation ketoacidosis, pancreatitis, alcoholic ketoacidosis, lactic acidosis, uremia, overdose of diabetic medication, and myocardial infarction. Ketoacidosis without hyperglycemia has been reported in pregnancy and in patients with prolonged vomiting or starvation. Euglycemic ketoacidosis has been reported in patients using SGLT-2i, where blood glucose may not be significantly elevated despite the ketoacidosis [25].

Management

**Figure 2:**
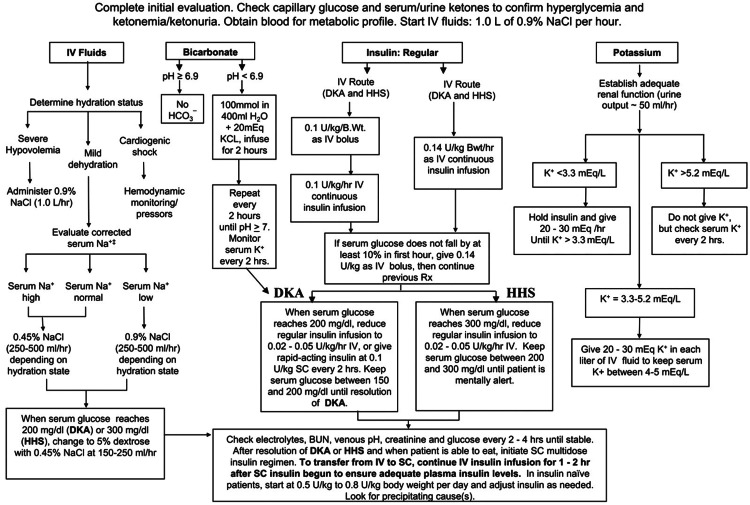
Flow chart for management of adult patients with diabetic ketoacidosis or hyperosmolar hyperglycemic state DKA: diabetic ketoacidosis; HHS: hyperosmolar hyperglycemic state Diabetic ketoacidosis is defined by blood glucose >250 mg/dL, arterial pH <7.3, bicarbonate <15 mEq/L, and moderate ketonuria or ketonemia; hyperosmolar hyperglycemic state is defined by serum glucose >600 mg/dL, arterial pH >7.3, bicarbonate >15 mEq/L, and minimal ketonuria or ketonemia. Initial fluid replacement is recommended at 15-20 mL/kg/h. Serum sodium should be corrected for hyperglycemia by adding 1.6 mEq/L to the measured sodium for every 100 mg/dL increase in glucose. Image from John Wiley and Sons and Copyright Clearance Center, with permission (license number: 6027620725584)

The treatment goals include rectification of dehydration, hyperglycemia, and hyperosmolality, electrolyte imbalance, ketonemia, as well as identification and treatment of precipitating risk factors. The American Diabetes Association's set of rules for the management of hyperglycemic emergencies is shown in Figure 2.

Initial resuscitation

Fluid Replacement

Initial management in both DKA and HHS is fluid resuscitation to restore intravascular volume, improve renal perfusion, and improve insulin sensitivity. DKA usually involves a fluid deficit of 5-7 liters, whereas HHS may exceed 8-12 liters due to prolonged osmotic diuresis. Isotonic saline (0.9% NaCl) at 15-20 mL/kg body weight is given in the first hour (about 1-1.5 liters in the first hour), with slower rates in case of the elderly, frail, or cardiac/renal-compromised patients. The use of Ringer’s lactate leads to faster resolution of DKA, shorter hospital stays, and lower incidence of hyperchloremic metabolic acidosis [30]. Once partial volume restoration is achieved, the fluid choice depends on the corrected serum sodium: half-normal saline (0.45% NaCl) is used if sodium is normal or elevated, and isotonic saline is continued if sodium is low. The estimated deficits should be restored within the first 24-48 h by fluid replacement. As soon as plasma glucose decreases to below 250 mg/dL, 5-10% dextrose should be used in addition to the normal saline to prevent hypoglycemia, while insulin is used to correct the ketonemia.

In case of HHS, the goal of the treatment is to replace almost 50% of the fluid deficit within the first 12 h, and the remainder in the next 12 h. An initial administration rate of 500-1,000 mL/h of normal saline should be maintained during the first 2-4 h. Fluid replacement alone reduces the plasma glucose level and the serum osmolality. A resulting increase in sodium level occurs; however, a hypotonic solution need not be used unless the decrease in osmolality is not adequate. An increase in serum sodium greater than 2.4 mmol/L for every 5.5 mmol/L fall in blood glucose suggests inadequate fluid replacement. This requires a higher infusion rate [31]. In case glucose and osmolality are not falling at the desired rate with adequate fluid replacement, 0.45% NaCl solution should be used. The osmolality should decrease at a rate of 3-8 mOsmol/kg per hour, sodium reduction should not exceed 10 mmol/L in 24 hours, and a decrease in glucose level should not exceed 90 mg/dL. Gradual correction of hyperosmolarity is critical to prevent cerebral edema, particularly in older patients. Ongoing monitoring of fluid balance, electrolytes, and hemodynamic status is essential, with adjustments based on urine output, mental status, and laboratory trends. Rapid correction of osmolality or glucose should be avoided to minimize complications like cerebral edema or osmotic demyelination [25,29,32].

Insulin Therapy

Insulin therapy in DKA and HHS aims to suppress ketogenesis, lower glucose levels, and correct metabolic derangements. In DKA, insulin is started immediately after diagnosis, along with fluid resuscitation. Care should be taken that the serum potassium level is at least 3.3 mEq/L at the time of initiation of insulin therapy. A fixed-rate continuous insulin infusion is initiated at 0.1 units/kg/hour. A short-acting insulin bolus may be used in severe cases, adjusted according to the patient’s requirements. Once the blood glucose level falls below 250 mg/dL, the rate of insulin infusion should be reduced to 0.05 units/kg/h and should be continued until resolution of ketoacidosis. Criteria for the resolution of ketoacidosis include plasma glucose < 200 mg/dL, venous pH > 7.3 and/or bicarbonate level > 18 mmol/L, and plasma ketone level < 0.6 mmol/L.

In case of TIDM patients who had been taking long-acting insulin before admission, basal insulin can be continued during administration of the intravenous (IV) insulin infusion. This will later help in the transition to a subcutaneous (SC) basal bolus regimen. In case of newly diagnosed patients with T1DM, basal insulin is initiated at 0.15-0.3 units/kg. After resolution of DKA and initiation of adequate oral intake, IV insulin can be discontinued, and a rapid-acting insulin is resumed with meals or initiated in the newly diagnosed patients. In case of patients who have not been on simultaneous IV insulin and SC basal insulin during treatment, the insulin infusion should be stopped at least 1-2 hours after administration of the SC insulin. If the oral intake is poor/insufficient, transition to variable rate insulin infusion along with glucose solutions is recommended. Premature discontinuation of IV insulin before normalization of all parameters is a common cause of DKA recurrence [33,34]. A total daily dose of 0.5-0.6 units/kg (split evenly between basal and bolus) is recommended for insulin-naive patients. This transition reduces glycemic variability and prevents ketosis recurrence, supported by glucose monitoring, dietary guidance, and patient education. In resource-limited settings, neutral protamine Hagedorn (NPH) insulin may be used as an interim option [29].

In case of HHS without acidosis (blood pH > 7.3, bicarbonate > 18 mmol/L) but with mild or moderate ketonemia (blood β-hydroxybutyrate ≥1.0 to <3.0 mmol/L or urine ketones <2+), insulin is initiated only after blood glucose stops decreasing, i.e., posthypovolemia correction. This prevents large osmotic shifts and subsequent neurologic complications [31]. The insulin infusion rate is kept low, at 0.05 units/kg/h. As glucose drops to or ~300 mg/dL, insulin rates are reduced and dextrose is added. Transition to SC insulin occurs once acidosis and hyperosmolarity resolve, with a 1-2 hour overlap to prevent rebound hyperglycemia [26,32]. The criteria for the resolution of HHS include overall serum osmolality (total and effective) < 300 mOsm/kg, blood glucose below 250 mg/dL, urine output > 0.5 mL/kg/h, and improvement in cognitive status [26].

Mixed DKA/HHS has been reported in more than a third of patients with hyperglycemic crisis. Criteria for the diagnosis of DKA/HHS include hyperosmolality (> 320 mOsm/kg), ketonemia (β-hydroxybutyrate ≥3.0 mmol/L or ketonuria ≥2+), and presence of acidosis (pH < 7.30, or bicarbonate < 18 mmol/L) [35]. DKA/HHS requires higher doses of insulin, similar to DKA (fixed rate insulin infusion initiated at 0.1 units/kg/h). IV fluids are initiated with the goal to achieve a positive balance of 3-6 L during the first 12 hours, and the remaining replacement in the following 12 hours. Complete resolution may take up to 72 hours [31]. Transition to SC insulin follows the same principles as DKA.

Electrolyte Correction

Potassium replacement: Total body potassium decreases in DKA/HHS, as a result of osmotic diuresis and concurrent hyperaldosteronism (renal loss) and vomiting (extrarenal loss). Cellular potassium moves into the extracellular space due to acidosis and insulin deficiency, masking the reduction in body potassium. Initiation of insulin therapy shifts the extracellular potassium back into the cells, decreasing serum potassium levels. A further fall in potassium level results from fluid restoration and increased renal potassium excretion. Despite initial hyperkalemia (often seen in DKA), insulin therapy without potassium supplementation can precipitate life-threatening hypokalemia. If initial serum potassium is <3.3 mEq/L, insulin therapy must be delayed to avoid the risk of lethal arrhythmias and respiratory muscle weakness until potassium is corrected to ≥3.3 mEq/L. This can be achieved by administering 20-30 mEq of potassium per hour. If potassium is between 3.3 and 5.2 mEq/L, concurrent potassium replacement with insulin is recommended (20-30 mEq per liter of IV fluids). If potassium is >5.2 mEq/L, replacement is withheld, but levels should be monitored closely [2,26].

Bicarbonate therapy: The use of sodium bicarbonate in DKA remains controversial. Routine bicarbonate therapy is not recommended unless the arterial pH is <6.9, as the risk of paradoxical central nervous system acidosis, hypokalemia, and delayed ketone clearance outweighs the benefits. In cases with severe acidosis (pH: <6.9), 100 mmol sodium bicarbonate diluted in 400 mL sterile water and 20 mEq potassium chloride over two hours is suggested, with close monitoring. In HHS, bicarbonate therapy is rarely needed due to the absence of significant ketoacidosis [26,29].

Phosphate replacement: Significant phosphate losses occur in DKA and HHS due to osmotic diuresis, leading to muscle weakness, respiratory depression, and cardiac dysfunction in severe cases. However, routine phosphate replacement is generally not recommended unless serum phosphate falls below 1.0 mg/dL or the patient develops cardiac dysfunction, anemia, or respiratory depression. Acute hypophosphatemia in a patient with pre-existing severe phosphate depletion can lead to rhabdomyolysis. If indicated, 20-30 mEq potassium phosphate can be added per liter of fluid, keeping in mind the risk of hypocalcemia and metastatic calcification [26,29].

Monitoring

DKA typically resolves within 10-18 hours and HHS within 9-11 hours, requiring close monitoring of vitals, fluid therapy, insulin dosing, urine output, and 2-4 hourly lab assessments of glucose, electrolytes, pH, bicarbonate, and anion gap [2]. Lack of frequent monitoring is the chief cause of hypoglycemia during insulin treatment. Many patients with hypoglycemia do not experience adrenergic manifestations of sweating, nervousness, fatigue, hunger, and tachycardia; thus, frequent blood glucose monitoring every 1-2 hours is mandatory [29]. During the acute phase of DKA, blood gases should be monitored every four hours to track the resolution of acidosis, indicated by rising bicarbonate and closing anion gap. In HHS, acid-base monitoring helps rule out overlapping DKA. These assessments guide insulin, fluid, and treatment adjustments. Strict four-hourly monitoring also allows early detection of complications like hypokalemia, worsening acidosis, or inadequate fluid resuscitation, which can be life-threatening if missed. Most clinical guidelines, including those from the American Diabetes Association (ADA) and the International Society for Pediatric and Adolescent Diabetes (ISPAD), emphasize such close monitoring to reduce morbidity and mortality [29,32].

Complications

Delayed Recognition Worsens Outcomes

Mortality is markedly increased when diagnosis is delayed or when fluid and electrolyte imbalances are not corrected appropriately [26]. Neurological complications such as cerebral edema are well-documented in DKA, particularly in pediatric patients, and significantly increase both morbidity and mortality [36]. In HHS, thromboembolic complications and severe dehydration-related vascular events are major contributors to poor outcomes [37]. Moreover, recurrent episodes of DKA or HHS are linked to increased risk of long-term organ dysfunction, including cardiovascular disease, chronic kidney disease, diabetic neuropathy, and retinopathy, due to prolonged metabolic instability and inflammation [24,38].

Complications During Management

In case of DKA, acute adverse outcomes of treatment include hypoglycemia with seizures, arrhythmias, and cardiovascular events. Clinicians should be aware that recurrent episodes of hypoglycemia might be associated with a state of hypoglycemia unawareness (loss of perception of warning symptoms of developing hypoglycemia), which may complicate diabetes management after resolution of hyperglycemic crises [2]. Many patients with hypoglycemia do not experience adrenergic manifestations of sweating, nervousness, fatigue, hunger, and tachycardia; thus, frequent blood glucose monitoring every 1-2 hours is mandatory in such patients [29]. Hypokalemia is the second most common complication during treatment of DKA and HHS using insulin. It occurs due to increased cellular potassium uptake in peripheral tissues [25]. Both conditions can result in acute kidney injury (AKI) due to hypovolemia and shock. Inadequate or delayed treatment can further worsen these outcomes. In addition, rhabdomyolysis can also appear due to muscle injury from metabolic derangement [39].

Chronic complications may develop due to recurrent episodes of DKA or HHS, leading to worsening diabetic complications like nephropathy, retinopathy, and neuropathy, due to prolonged periods of hyperglycemia and systemic inflammation. Cardiovascular events like myocardial infarction and stroke may develop. Moreover, recurrent DKA episodes are linked to psychological issues, poor glycemic control, and increased risk of mortality. Therefore, early recognition, prompt correction of fluid deficits, electrolyte imbalances, and addressing the underlying cause are key to preventing both acute and long-term complications in DKA and HHS [13,26,32].

Transition to subcutaneous insulin

The transition from IV to SC insulin in the management of DKA and HHS marks a pivotal shift from acute resuscitation to long-term glycemic stabilization. This step must be approached with clinical precision to avoid rebound hyperglycemia or relapse of ketosis. Initiation of subcutaneous insulin is recommended only when blood glucose levels have fallen to <200 mg/dL, and the patient demonstrates clear resolution of ketoacidosis, indicated by normalized anion gap, pH of >7.3, bicarbonate of >15-18 mEq/L, and absence of ketonemia [26]. To ensure therapeutic continuity, IV insulin should overlap with subcutaneous insulin for 1-2 hours to compensate for the delayed action of long-acting analogs like glargine or detemir [32].

Prevention and education

Empowering Patients Through Education

Effective prevention of DKA and HHS starts with structured patient education, emphasizing consistent insulin use, regular glucose monitoring, and early recognition of hyperglycemia and ketosis. Diabetes is a chronic condition where the patient has to be actively involved in the therapy. Self-management of the condition is essential, as lifestyle changes, timely meals and medication, and exercise are a part of the treatment and have to be carried out by the patients themselves. The patients must also be aware of the complications as well as the management of these complications. Persons living with the patient (spouse and other family members) should also be aware of the lifestyle interventions required and the management of acute complications [40]. Individualized education significantly reduces recurrent DKA rates [32]. The effect of therapeutic patient education in diabetic and obese patients using different techniques has shown significant improvement in biomedical outcomes by increasing the standard of self-management [41]. Education should not be limited to initial diagnosis but reinforced at regular intervals, especially after any acute hospitalization.

Sick-Day Rules

During illness, infection, or stress, endogenous hormones like cortisol and catecholamines increase insulin resistance and hepatic glucose output, escalating the risk of metabolic decompensation. Patients should follow clearly defined "sick-day guidelines," such as never omitting insulin, checking blood glucose and ketones more frequently, and maintaining adequate hydration. Novel tools like smartphone-based reminders or sick-day mobile applications are increasingly being explored to guide real-time insulin adjustment and communication with care teams during illness episodes [29].

Nutrition and Lifestyle: A Foundation for Stability

Long-term prevention of DKA and HHS relies on healthy lifestyle habits, including a balanced low-glycemic diet, adequate hydration, and regular physical activity to improve insulin sensitivity. For high-risk patients, community follow-up and peer counselling can help reduce rehospitalizations [25]. Tailored interventions considering cultural dietary patterns and socioeconomic status can further enhance adherence and effectiveness. The involvement of household members, especially the spouse and children, has been found to increase adherence to lifestyle interventions in Indian households [40]. 

Prognosis

The clinical outcome of DKA and HHS largely hinges on the speed and appropriateness of medical intervention. With prompt and standardized treatment protocols, the prognosis of DKA is generally favorable, with reported mortality rates ranging between 1% and 5%, most deaths occurring in the elderly or those with severe comorbid conditions [26]. In contrast, HHS carries a significantly higher mortality rate, approaching 15-20%, due to its frequent occurrence in older individuals, who often present with delayed diagnosis, profound dehydration, and multiple comorbidities [29].

Importance of Long-Term Glycemic Control

Long-term prognosis also depends on addressing the underlying cause, ensuring patient education, and maintaining consistent glycemic control to prevent recurrence. Psychosocial support, especially in patients with poor adherence or limited access to care, plays a key role in reducing readmissions and mortality. This, again, can be increased through education of the family as well as the patient. The integration of multidisciplinary care teams, including endocrinologists, diabetes educators, and primary care physicians, has been shown to improve long-term survival and reduce complications [26,29,32]. The prognosis of HHS patients depends on dehydration severity, comorbidities, and age; those with a history of multiple HHS episodes face a significantly higher risk of posthospitalization mortality. A recent study reported that after adjustment for age, sex, selected comorbidities, and monthly income, the mortality hazard ratio was higher in subjects who had two or more episodes of hyperglycemic crisis in patients with diabetes without HHS [4].

Transforming challenges: from crisis to control

The management of chronic uncontrolled diabetes complicated by DKA and HHS represents not just a medical challenge but also an opportunity to redefine long-term care and patient empowerment. While these acute metabolic crises are life-threatening, their occurrence often signals gaps in chronic disease management, education, and healthcare access. With timely diagnosis, evidence-based treatment protocols, and compassionate multidisciplinary care, DKA and HHS are both treatable and largely preventable. True success in diabetes care extends beyond acute intervention-it requires empowering patients through structured follow-up, lifestyle support, psychosocial care, and equitable access to modern treatments. By transforming crises into turning points, we can restore metabolic stability, prevent recurrence, and improve both survival and quality of life. Further research is needed to refine treatment protocols and explore innovative therapies.

## Conclusions

The management of chronic uncontrolled diabetes complicated by DKA or HHS represents a medical challenge as well as an opportunity to redefine long-term care and patient empowerment. The occurrence of these life-threatening acute metabolic crises often signals gaps in chronic disease management, education, and healthcare access. DKA in undiagnosed T1DM typically presents with abdominal pain, nausea, vomiting, Kussmaul respiration, fruity breath odor, and dehydration. The onset of symptoms is rapid, often preceded by symptoms of classic hyperglycemia (polyuria, polydipsia, and weight loss). HHS usually affects older individuals and develops gradually over days or weeks, often triggered by infection or illness. Neurological deficits such as confusion, vision loss, or seizures are common due to hyperosmolarity. Patients frequently present with signs of dehydration. HHS carries a high thromboembolic risk from increased blood viscosity. The diagnosis of DKA requires the presence of hyperglycemia, ketonemia, and metabolic acidosis, with an elevated anion gap. HHS is identified by severe hyperglycemia, hyperosmolality, minimal ketonemia, and absence of acidosis. Pseudohyponatremia, misleading ketone tests, and unrecognized osmotic contributors like alcohol can complicate the diagnosis. Serum potassium and phosphate monitoring is critical, as levels may initially appear normal or elevated, masking total body depletion. Management involves aggressive fluid resuscitation, insulin therapy, and electrolyte correction. Fluid deficits are larger in HHS, necessitating careful, gradual rehydration to prevent cerebral edema. Insulin therapy in DKA starts immediately but is delayed in HHS until after volume restoration. Potassium replacement is essential during insulin therapy to avoid hypokalemia.

Recurrent DKA or HHS episodes increase the risk of long-term organ damage, including cardiovascular disease, chronic kidney disease, and neuropathy. Monitoring vital signs, electrolytes, and acid-base status every few hours is critical to prevent complications like hypoglycemia or hypokalemia. Delayed recognition worsens outcomes, with cerebral edema in DKA and thromboembolic events in HHS as major mortality contributors. Transition to subcutaneous insulin requires careful overlap to prevent relapse. Prevention strategies include patient education, sick-day guidelines, lifestyle modifications, and structured follow-up to ensure glycemic control and reduce recurrence. With timely diagnosis, evidence-based treatment protocols, and compassionate multidisciplinary care, DKA and HHS are both treatable and largely preventable. Diabetes care requires empowering patients through structured follow-up, lifestyle support, psychosocial care, and equitable access to modern treatments. By transforming crises into turning points, we can restore metabolic stability, prevent recurrence, and improve both survival and quality of life. 

## References

[1] Goyal R, Singhal M, Jialal I (2023). Type 2 Diabetes. NBK513253.

[2] Fayfman M, Pasquel FJ, Umpierrez GE (2017). Management of hyperglycemic crises: diabetic ketoacidosis and hyperglycemic hyperosmolar state. Med Clin North Am.

[3] Karges B, Rosenbauer J, Holterhus PM (2015). Hospital admission for diabetic ketoacidosis or severe hypoglycemia in 31,330 young patients with type 1 diabetes. Eur J Endocrinol.

[4] Huang CC, Weng SF, Tsai KT (2015). Long-term mortality risk after hyperglycemic crisis episodes in geriatric patients with diabetes: a national population-based cohort study. Diabetes Care.

[5] Praveen PA, Hockett CW, Ong TC (2021). Diabetic ketoacidosis at diagnosis among youth with type 1 and type 2 diabetes: results from SEARCH (United States) and YDR (India) registries. Pediatr Diabetes.

[6] Gregg EW, Williams DE, Geiss L (2014). Changes in diabetes-related complications in the United States. N Engl J Med.

[7] Seth P, Kaur H, Kaur M (2015). Clinical profile of diabetic ketoacidosis: a prospective study in a tertiary care hospital. J Clin Diagn Res.

[8] SP B, Pejaver R, K R, V S, MT SB (2015). Clinical profile and outcome of diabetic ketoacidosis in a tertiary care hospital in South India. Int J Contemp Pediatr.

[9] Kiran R, Saroch A, Pannu AK, Sharma N, Dutta P, Kumar M (2022). Clinical profile and outcomes of diabetic ketoacidosis during the COVID-19 pandemic in North India. Trop Doct.

[10] Gaikwad V, Deshmukh JK, Deshmukh P, Takalkar AA (2020). Clinical features and risk factors for diabetic ketoacidosis in type 1 diabetes mellitus at tertiary care centre, Maharashtra, India. Int J Adv Med.

[11] Laffel L (1999). Ketone bodies: a review of physiology, pathophysiology and application of monitoring to diabetes. Diabetes Metab Res Rev.

[12] Miles JM, Haymond MW, Nissen SL, Gerich JE (1983). Effects of free fatty acid availability, glucagon excess, and insulin deficiency on ketone body production in postabsorptive man. J Clin Invest.

[13] McGarry JD, Foster DW (1980). Effects of exogenous fatty acid concentration on glucagon-induced changes in hepatic fatty acid metabolism. Diabetes.

[14] McGarry JD, Foster DW (1980). Regulation of hepatic fatty acid oxidation and ketone body production. Annu Rev Biochem.

[15] Umpierrez GE, Davis GM, ElSayed NA (2024). Hyperglycaemic crises in adults with diabetes: a consensus report. Diabetologia.

[16] Kitabchi AE, Umpierrez GE, Murphy MB, Barrett EJ, Kreisberg RA, Malone JI, Wall BM (2001). Management of hyperglycemic crises in patients with diabetes. Diabetes Care.

[17] Delaney MF, Zisman A, Kettyle WM (2000). Diabetic ketoacidosis and hyperglycemic hyperosmolar nonketotic syndrome. Endocrinol Metab Clin North Am.

[18] Nyenwe EA, Loganathan RS, Blum S (2007). Active use of cocaine: an independent risk factor for recurrent diabetic ketoacidosis in a city hospital. Endocr Pract.

[19] Davis SN, Umpierrez GE (2007). Diabetic ketoacidosis in type 2 diabetes mellitus--pathophysiology and clinical presentation. Nat Clin Pract Endocrinol Metab.

[20] Garg SK, Walker AJ, Hoff HK, D'Souza AO, Gottlieb PA, Chase HP (2004). Glycemic parameters with multiple daily injections using insulin glargine versus insulin pump. Diabetes Technol Ther.

[21] Nyenwe EA, Kitabchi AE (2011). Evidence-based management of hyperglycemic emergencies in diabetes mellitus. Diabetes Res Clin Pract.

[22] Diabetes Control and Complications Trial Research Group (1995). Implementation of treatment protocols in the diabetes control and complications trial. Diabetes Care.

[23] Wachtel TJ, Tetu-Mouradjian LM, Goldman DL, Ellis SE, O'Sullivan PS (1991). Hyperosmolarity and acidosis in diabetes mellitus: a three-year experience in Rhode Island. J Gen Intern Med.

[24] Barski L, Eshkoli T, Brandstaetter E, Jotkowitz A (2019). Euglycemic diabetic ketoacidosis. Eur J Intern Med.

[25] Kitabchi AE, Umpierrez GE, Miles JM, Fisher JN (2009). Hyperglycemic crises in adult patients with diabetes. Diabetes Care.

[26] Gerich JE, Martin MM, Recant L (1971). Clinical and metabolic characteristics of hyperosmolar nonketotic coma. Diabetes.

[27] Sheikh-Ali M, Karon BS, Basu A (2008). Can serum beta-hydroxybutyrate be used to diagnose diabetic ketoacidosis?. Diabetes Care.

[28] Glaser N, Barnett P, McCaslin I (2001). Risk factors for cerebral edema in children with diabetic ketoacidosis. N Engl J Med.

[29] Umpierrez G, Korytkowski M (2016). Diabetic emergencies-ketoacidosis, hyperglycaemic hyperosmolar state and hypoglycaemia. Nat Rev Endocrinol.

[30] Li S, Mikhael B, van Zyl DG (2025). Choice of intravenous fluid for resuscitation in diabetic ketoacidosis. N Engl J Med.

[31] Mustafa OG, Haq M, Dashora U, Castro E, Dhatariya KK (2023). Management of hyperosmolar hyperglycaemic state (HHS) in adults: an updated guideline from the Joint British Diabetes Societies (JBDS) for inpatient care group. Diabet Med.

[32] Wolfsdorf JI, Glaser N, Agus M (2018). ISPAD clinical practice consensus guidelines 2018: diabetic ketoacidosis and the hyperglycemic hyperosmolar state. Pediatr Diabetes.

[33] Kitabchi AE, Umpierrez GE, Murphy MB, Kreisberg RA (2006). Hyperglycemic crises in adult patients with diabetes: a consensus statement from the American Diabetes Association. Diabetes Care.

[34] Kitabchi AE, Nyenwe EA (2006). Hyperglycemic crises in diabetes mellitus: diabetic ketoacidosis and hyperglycemic hyperosmolar state. Endocrinol Metab Clin North Am.

[35] Jiang DH, Herrin J, Van Houten HK, McCoy RG (2023). Evaluation of high-deductible health plans and acute glycemic complications among adults with diabetes. JAMA Netw Open.

[36] Pasquel FJ, Umpierrez GE (2014). Hyperosmolar hyperglycemic state: a historic review of the clinical presentation, diagnosis, and treatment. Diabetes Care.

[37] Umpierrez GE, Kelly JP, Navarrete JE, Casals MM, Kitabchi AE (1997). Hyperglycemic crises in urban blacks. Arch Intern Med.

[38] Sacchetta L, Chiriacò M, Nesti L (2022). Synergistic effect of chronic kidney disease, neuropathy, and retinopathy on all-cause mortality in type 1 and type 2 diabetes: a 21-year longitudinal study. Cardiovasc Diabetol.

[39] Wang LM, Tsai ST, Ho LT, Hu SC, Lee CH (1994). Rhabdomyolysis in diabetic emergencies. Diabetes Res Clin Pract.

[40] Saxena I, Kumar M, Kannoujia BL, Aishwarya R, Sen A (2025). Investigating the causes of non-adherence to recommended lifestyle modifications among Indian patients with newly diagnosed type 2 diabetes mellitus or hypertension. Cureus.

[41] Correia JC, Waqas A, Huat TS, Gariani K, Jornayvaz FR, Golay A, Pataky Z (2022). Effectiveness of therapeutic patient education interventions in obesity and diabetes: a systematic review and meta-analysis of randomized controlled trials. Nutrients.

